# Orphan nuclear receptor ERRγ is a key regulator of human fibrinogen gene expression

**DOI:** 10.1371/journal.pone.0182141

**Published:** 2017-07-27

**Authors:** Yaochen Zhang, Don-Kyu Kim, Yan Lu, Yoon Seok Jung, Ji-min Lee, Young-Hoon Kim, Yong Soo Lee, Jina Kim, Bedair Dewidar, Won-IL Jeong, In-Kyu Lee, Sung Jin Cho, Steven Dooley, Chul-Ho Lee, Xiaoying Li, Hueng-Sik Choi

**Affiliations:** 1 National Creative Research Initiatives Center for Nuclear Receptor Signals and Hormone Research Center, School of Biological Sciences and Technology, Chonnam National University, Gwangju, Republic of Korea; 2 Shanghai Institute of Endocrinology and Metabolism, Shanghai Key Laboratory for Endocrine Tumors, Rui-Jin Hospital, Shanghai Jiao Tong University School of Medicine, Shanghai, China; 3 Korea Research Institute of Bioscience and Biotechnology, Daejeon, Republic of Korea; 4 New Drug Development Center, Daegu-Gyeongbuk Medical Innovation Foundation, Daegu, Korea; 5 Department of Medicine II, Medical Faculty Mannheim, Heidelberg University, Mannheim, Germany; 6 Graduate School of Medical Science and Engineering, Korea Advanced Institute of Science and Technology, Daejeon, Republic of Korea; 7 Department of Internal Medicine, School of Medicine, Kyungpook National University, Daegu, Korea; 8 Leading-edge Research Center for Drug Discovery and Development for Diabetes and Metabolic Disease, Kyungpook National University Hospital, Daegu, Korea; Vall d'Hebron Institut de Recerca, SPAIN

## Abstract

Fibrinogen, 1 of 13 coagulation factors responsible for normal blood clotting, is synthesized by hepatocytes. Detailed roles of the orphan nuclear receptors regulating fibrinogen gene expression have not yet been fully elucidated. Here, we identified estrogen-related receptor gamma (ERRγ) as a novel transcriptional regulator of human fibrinogen gene expression. Overexpression of ERRγ specially increased fibrinogen expression in human hepatoma cell line. Cannabinoid receptor types 1(CB1R) agonist arachidonyl-2'-chloroethylamide (ACEA) up-regulated transcription of fibrinogen via induction of ERRγ, whereas knockdown of ERRγ attenuated fibrinogen expression. Deletion analyses of the fibrinogen γ (FGG) gene promoter and ChIP assays revealed binding sites of ERRγ on human fibrinogen γ gene promoter. Moreover, overexpression of ERRγ was sufficient to increase fibrinogen gene expression, whereas treatment with GSK5182, a selective inverse agonist of ERRγ led to its attenuation in cell culture. Finally, fibrinogen and ERRγ gene expression were elevated in liver tissue of obese patients suggesting a conservation of this mechanism. Overall, this study elucidates a molecular mechanism linking CB1R signaling, ERRγ expression and fibrinogen gene transcription. GSK5182 may have therapeutic potential to treat hyperfibrinogenemia.

## Introduction

Obesity is frequently associated with elevated risk for cardiovascular disease (CVD) [1]. In addition, accumulating evidence indicates that elevated blood fibrinogen level is a risk factor for the development of CVD [2, 3]. Fibrinogen (Factor I) is a 340 kDa glycoprotein synthesized in liver by hepatocytes [4]. The three chains of fibrinogen (Aα, Bβ, and γ) are encoded by different genes, *FGA*, *FGB*, and *FGG*, respectively [5]. They form an elongated molecule that binds to a second identical molecule by disulfide bonds, forming the homodimeric fibrinogen molecule that circulates in the blood [6–8]. Hyperfibrinogenemia and hypofibrinolysis are associated with a hypercoagulable state that causes accumulation of fibrin, increasing the risk for thrombotic events and CVD [9]. In both adults and children, obesity is characterized by various derangements in key components of the hemostatic system, including the presence of hyperfibrinogenemia [10], which increases thrombus fibrin content, accelerates fibrin formation, and increases fibrin network stability. Hyperfibrinogenemia also increases thrombus resistance to tenecteplase-induced thrombolysis [11]. Down-regulation of fibrinogen expression may prevent CVD. The three fibrinogen genes are targets of several nuclear receptors. RAR-related orphan receptor alpha (RORα) regulates *FGB* expression in human hepatoma cells and mouse liver [12]. Endogenous bile acids are known as ligands for the nuclear receptor farnesoid X receptor (FXR), and all three fibrinogen subunits are induced by FXR in response to FXR ligands, suggesting that bile acids and FXR modulate fibrinolytic activity [13]. In addition, the nuclear receptor coactivator 2 (NCoA-2) is a positive regulator of *FGB* transcription, and sequestration of NCoA-2 by PPARα is a molecular mechanism by which PPARα agonists negatively regulate fibrinogen-β [14]. However, regulation of fibrinogen gene expression by members of the nuclear receptor superfamily remains largely uncharacterized.

The estrogen receptor–related receptor subfamily consists of three members, ERRα, β, and γ (NR3B1-3), which bind to both classic estrogen response elements (ERE) and extended half-site core sequences (TNAAGGTCA; ERR response element or ERRE) as either monomers or dimers [15]. Structural studies indicate that ERRγ is constitutively active in the absence of endogenous ligands, whereas small-molecule ligands can further activate or repress ERRγ transactivation [16–19]. The ligand-independent transcriptional activity of ERRγ depends on nuclear receptor co-regulators, such as NCoA-2, PGC-1α, receptor-interacting protein 140 (RIP140), and small heterodimer partner (SHP), and these co-regulators are involved in the regulation of liver metabolism [20–24]. ERRs are expressed in tissues with high metabolic demand and are regulated by the peripheral circadian clock in key metabolic tissues such as white and brown adipose tissues, muscle, and liver [25]. In a previous study, we showed that ERRγ regulates glucose metabolism by modulating phosphoenolpyruvate carboxykinase 1 (PEPCK) and glucose-6-phosphatase (G6Pase) gene expression, rate-limiting enzymes in glucose production [26, 27]. We also found that ERRγ participates in regulating pyruvate dehydrogenase kinase 4 (PDK4) gene expression and is a novel transcriptional regulator of phosphatidic acid phosphatase [28, 29]. Recently, we demonstrated that ERRγ is a transcriptional regulator of *CYP7A1* gene expression and increases bile acid synthesis [30] and hepcidin expression [31]. We also showed that ERRγ controls hepatic CB1R–mediated *CYP2E1* expression and oxidative liver injury upon alcohol use [32]. However, the role of ERRγ in liver metabolism is still not clear.

The endocannabinoid system comprises cannabinoid receptor types 1 and 2 (CB1R and CB2R). CB1R is expressed at high levels in the brain, but is also present at much lower concentrations in peripheral tissues, whereas CB2R is expressed predominantly in immune and hematopoietic cells [33]. Endocannabinoids (ECs) acting via CB1R play important roles in the control of body weight and energy homeostasis. Animal studies and clinical investigations in patient have shown that, in the obese state, the endocannabinoid system is hyperactivated because of impaired energy balance [34–36]. In obese or hyperglycemic type 2 diabetic patients, circulating levels of N-Arachidonoylethanolamine (AEA) and 2-Arachidonoylglycerol (2-AG) are elevated, and high levels of 2-AG are found in visceral adipose tissue [35, 37, 38]. Several studies indicate that inhibition of CB1R activity in peripheral tissues contributes to the metabolic benefits [36, 39], raising the possibility that selective targeting of peripheral CB1R could be used to treat metabolic syndrome. This concept is supported by recent studies, using a peripherally restricted neutral CB1R antagonist, AM6545[40], or a peripheral CB1R inverse agonist, JD5037 [41], in mice with high-fat diet–induced obesity (DIO). Our previous study revealed that activation of CB1R disrupts hepatic insulin receptor signaling via CREBH-mediated induction of *Lipin1* gene expression [42]. In another study, we identified a novel mechanism of regulation of hepatic bile acid metabolism by alcohol via CB1R-mediated activation of ERRγ [30]. Together, these findings suggest that blocking the CB1R signaling pathway can restore hepatic metabolic homeostasis.

In this study, we identified the nuclear receptor ERRγ as a transcriptional regulator of hepatic fibrinogen gene expression. An increase in hepatic ERRγ gene expression led to the induction of fibrinogen, whereas ablation of hepatic ERRγ gene expression abolished this induction. Activation of hepatic CB1R signaling induced ERRγ-mediated transcription of the fibrinogen gene. GSK5182, a selective ERRγ inverse agonist, decreased ACEA-mediated induction of fibrinogen. Based on these findings, inhibition of the transcriptional activity of ERRγ by an inverse agonist may have the potential to ameliorate hyperfibrinogenemia.

## Materials and methods

### Clinical samples

Obese subjects were screened with physical examination for fatty liver (age range from 36 to 65 years with mean age of 56.06) in China from April to August 2011. BMI was calculated as weight (in kilograms) divided by the square of the height (in meters). Those who drank 140 or 70 g/week of alcohol (for men or women, respectively), at the time or in the previous 6 months of investigation were excluded from the study. HBV- or HCV-infected subjects were also excluded. For analyses of circulating fibrinogen levels, blood samples were collected from 30 obese patients with BMI >25 and 30 aged-matched normal subjects with BMI < 25. For analysis of hepatic ERRγ and fibrinogen mRNA expression, liver biopsy was performed in 16 obese subjects and 14 healthy subjects, who donated their partial livers for liver transplantation. Immunohistochemical staining was performed in liver tissue of 3 randomly selected patients with biopsy proven NASH. The baseline demographic characteristics of human subjects are described in S2 Table. Patient information was not revealed in this study, and the data were analyzed blindly. Acquisition and handling of patient materials were approved by the Ethical Committees of Shanghai Jiao Tong University School of Medicine and Medical Faculty of Mannheim at Heidelberg University (Az: 2007-011N-MA). We confirm that this study was conducted in accordance with the Declaration of Helsinki. None of the transplant donors were from a vulnerable population and all donors or next of kin provided written informed consent that was freely given.

### Chemicals and plasmids

GSK5182 was synthesized as previously described with slight modifications[16]. ACEA and AM251 were purchased from Tocris Bioscience. The promoters of human *FGA* (-1.7 kb/+243 bp), *FGB* (-1.7 kb/+258 bp), and *FGG* (-1.5 kb/+233 bp, -1.2 kb/+233 bp, and -0.8 kb/+233 bp) were cloned into the *Xho*I/*Mlu*I sites of the PGL3-basic vector. These reporter plasmids were confirmed by DNA sequencing. Expression vectors for FLAG-ERRα, FLAG-ERRβ, and FLAG-ERRγ were described previously[27]. A mutation was introduced into the ERRE of the *FGG* promoter by site-directed mutagenesis (Stratagene, La Jolla, CA, USA), and the resultant construct (MT ERRE-luc) was confirmed by DNA sequencing.

### Recombinant adenovirus

Ad-GFP, Ad-FLAG-ERRγ, Ad-USi and Ad-shERRγ were described previously [26]. All viruses were purified using CsCl_2_ or an Adeno-X maxi purification kit (Clontech, Palo Alto, CA, USA). Adenoviral infections in cells were described previously [30].

#### Cell culture and transient transfection assay

293T, HepG2 and Huh7 cells were maintained as described previously [28]. The cells were used for experiments at 80% confluence. Transient transfections were conducted as described previously [28]. Luciferase activity was normalized to β-galactosidase activity.

### Measurement of fibrinogen level

Fibrinogen was extracted from cell culture medium and mouse blood, and its levels were determined using the Fibrinogen SimpleStep ELISA Kit (Abcam, Cambridge, MA, USA). Fibrinogen levels in human patients were analyzed by MILLIPLEX MAP Human Cardiovascular Disease (Acute Phase) Magnetic Bead Panel 3 (HCVD3MAG-67K, Milliplex Map, EMD Millipore, Billerica, MA, USA).

### Real-time PCR and Western blot analysis

RT–PCR and western blot analysis were performed as described previously [28]. The following primary antibodies were used for the immunoblotting assay: β-actin (AbFrontier, Seoul, Korea), ERRγ (Perseus Proteomics, Tokyo, Japan), and fibrinogen (Dako, Carpinteria,CA, USA). All primer sequences are described in S1 Table.

### Histological analysis

Liver tissues from patients with liver disease were fixed in 4% formaldehyde and embedded in paraffin. The slides were deparaffinized in xylene and rehydrated in a dilution series of graded ethanol to distilled water. Antigen retrieval was performed by microwave treatment in EDTA buffer (1 mmol/L, pH 8.0) for 10 minutes. The slides were incubated with 3% H_**2**_O_**2**_ for 30 minutes at room temperature. After washing with phosphate-buffered saline three times, slides were incubated with primary antibodies (1. ERRγ: Abcam; ab131593; 1:100 **or** 2. Fibrinogen: Abcam; ab58207; 1:100) at 4°C overnight. The next day, slides were washed with phosphate-buffered saline three times, followed by incubation with anti-mouse antibody HRP (Dako; 1:200) for 1 hour at room temperature. Color was developed with Diaminobenzidine (Sigma). Immunoreactivity was visualized under light microscopy.

#### ChIP assay

The ChIP assay was performed using a Chromatin Immunoprecipitation Assay Kit (Upstate Biotechnology, Lake Placid, NY, USA). Immunoprecipitation was performed using ERRγ antibody (Perseus Proteomics, Tokyo, Japan) or IgG (as a negative control). After recovering the DNA, polymerase chain reaction (PCR) was performed using primers encompassing the FGG promoter region. All primer sequences are described in S1 Table.

#### Statistics

Values are expressed as means ± standard error. Statistical significance was calculated using the unpaired Student’s *t*-test and one-way analysis of variance. Differences were considered significant at *p*<0.05.

## Results

### Overexpression of ERRγ induces fibrinogen gene expression

**Fig 1:**
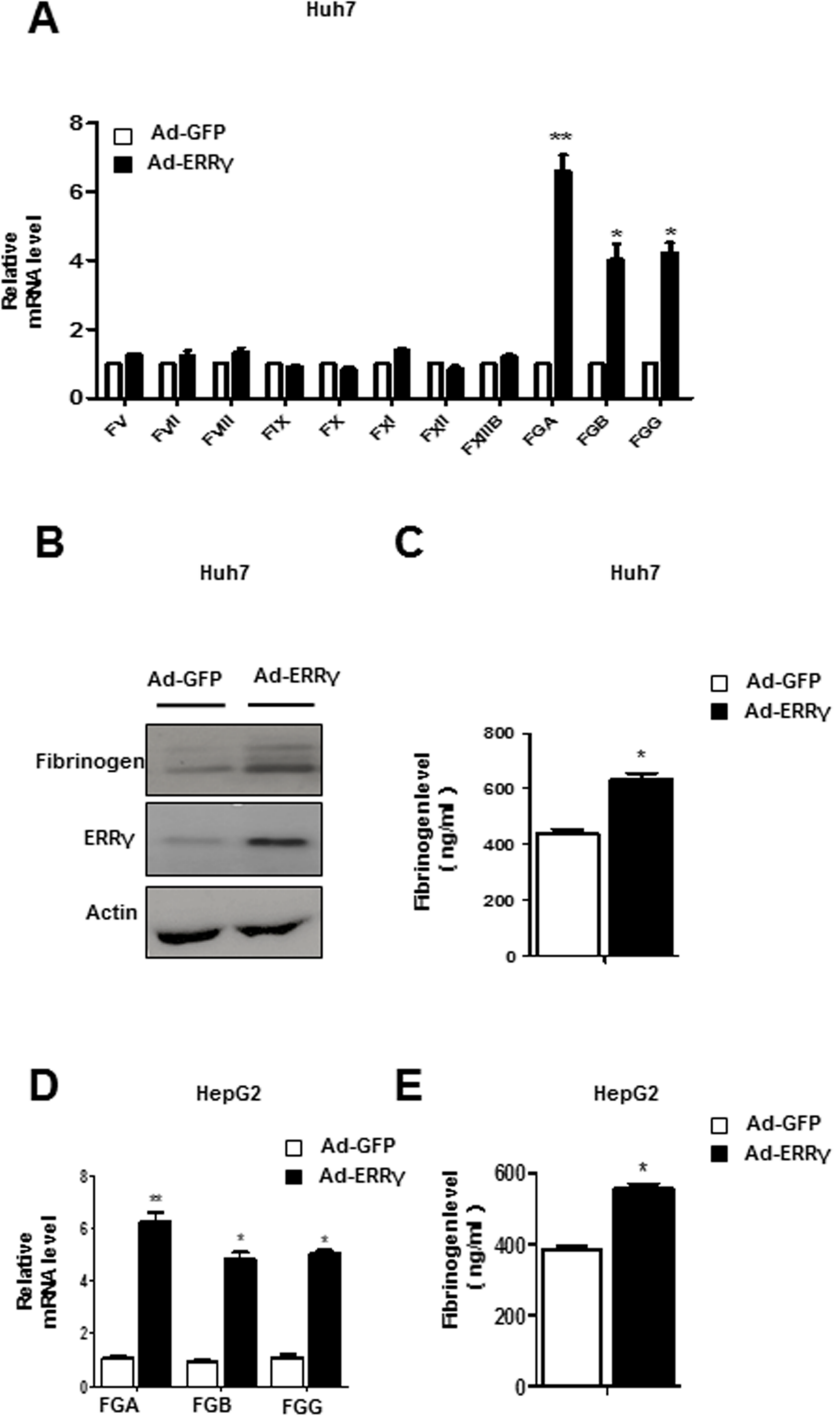
Overexpression of ERRγ induces fibrinogen gene expression. **(A-C)** Huh7 cells were infected with Ad-GFP (control) or Ad-ERRγ. Total RNA and protein were isolated and used for qPCR (A) and western blot analyses (B). Cell culture media were collected to determine fibrinogen levels (C). Western blot images were cropped with a black cropping line. All gels for Western blot analysis were run under the same experimental conditions. Full uncropped blots are available as S1 Fig. **(D-E)** HepG2 cells were infected with Ad-GFP (control) or Ad-ERRγ. Total RNA were isolated and used for qPCR (D) Cell culture supernatants were collected to determine fibrinogen levels (E)* *p*<0.05, ** *p*<0.01. All data are representative of at least three independent experiments. Error bars show SEM.

To define a functional link between ERRγ and coagulation factors gene expression, we overexpressed ERRγ using an adenovirus expressing ERRγ (Ad-ERRγ). Specifically, overexpression of ERRγ by Ad-ERRγ increased fibrinogen mRNA level in Huh7 and HepG2 cells **(Fig 1A and 1D)**. A similar increase in fibrinogen protein level was also found in Ad-ERRγ infected Huh7 cell **(Fig 1B)**. By contrast, overexpression of ERRγ had no significant effect on other coagulation factors in Huh7 cell line **(Fig 1A)**. We also collected cell culture supernatant of Huh7 and HepG2 cells to measure levels of secreted fibrinogen, which were significantly increased upon overexpression of ERRγ **(Fig 1C and 1E)**. Taken together, these results suggest that ERRγ can induce fibrinogen gene expression.

### Activation of the hepatic CB1 receptor induces fibrinogen expression

**Fig 2:**
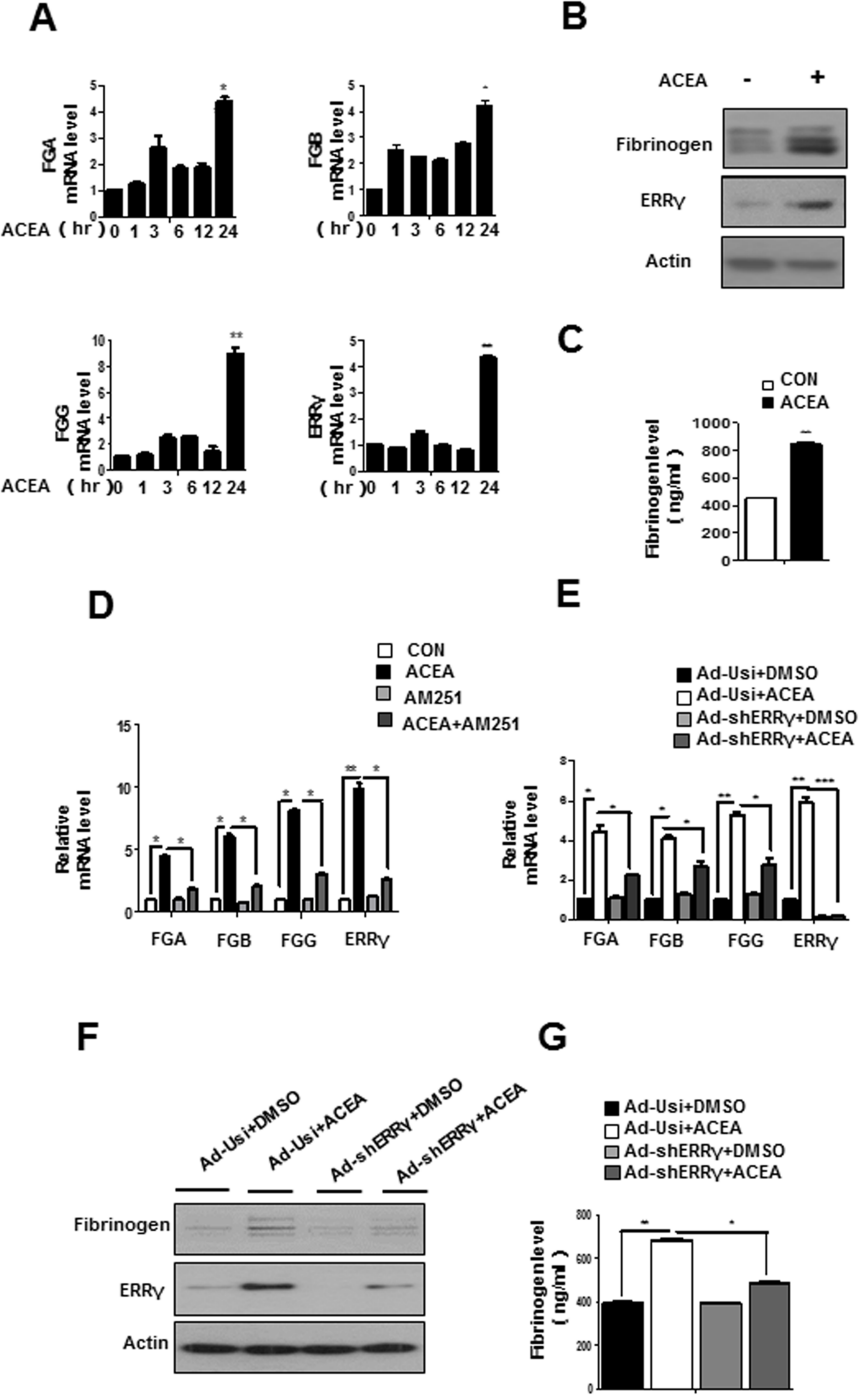
Knockdown of ERRγ attenuates ACEA-mediated induction of fibrinogen. **(A)** ACEA-mediated induction of fibrinogen expression. Huh7 cells were treated with ACEA (10 μM) for the indicated time periods. Total RNAs were extracted for qPCR analyses. **(B-C)** Huh7 cells were treated with ACEA (10 μM) for 24 h. Total protein was extracted for western blotting (B). Cell culture media were collected to determine fibrinogen levels (C). * *p*<0.05, ** *p*<0.01. All data are representative of at least three independent experiments. Error bars show SEM. **(D)** Huh7 cells were treatment with ACEA in the continued presence or absence of AM251 for 24 h. qPCR were performed to measure mRNA levels. * *p*<0.05. ** *p*<0.01. **(E-G)** qPCR (E) and western blot (F) analysis showing mRNA and protein levels of ERRγ, FGA, FGB, and FGG in Huh7 cells. Huh7 cells were infected with Ad-Usi or Ad-shERRγ for 48 h, followed by treatment with ACEA (10 μM). Cell culture media were collected to determine fibrinogen levels (G).Western blot images were cropped with a black cropping line. All gels for Western blot analysis were run under the same experimental conditions. Full uncropped blots are available as S1 and S2 Figs.

Knowing that the endocannabinoid system is hyperactivated in the obesity stage[35] and due to our previous study, where we showed that CB1R induces expression of ERRγ and its effect on modulating hepatic bile acid metabolism by alcohol [30], we speculated that CB1R might also involve in regulating fibrinogen expression in liver. We performed time course experiments to examine the induction of fibrinogen and ERRγ expression by ACEA, agonist of CB1R. ACEA significantly increased fibrinogen and ERRγ mRNA levels in Huh7 **(Fig 2A)**, with maximal levels at 24 h. Consistent with the change in mRNA levels, fibrinogen and ERRγ protein levels were also elevated during ACEA treatment of Huh7 cells **(Fig 2B)**. Moreover, ACEA treatment elevated fibrinogen level in the cell culture medium of Huh7 cells **(Fig 2C)**.Then, AM251, a selective inverse agonist of CB1R, antagonized the effects of ACEA mediated induction of ERRγ and fibrinogen mRNA levels in Huh7 cells **(Fig 2D)**. These results demonstrate that activation of the hepatic CB1 receptor increases ERRγ and fibrinogen expression at the mRNA and protein levels.

To functionally strengthen the link between CB1 receptor signaling and ERRγ in hepatic regulation of fibrinogen gene expression, we examined the effect of ERRγ knockdown by adenoviral overexpression of ERRγ shRNA (Ad-shERRγ). ACEA treatment increased ERRγ and fibrinogen mRNA levels in Huh7 cells, whereas fibrinogen expression was inhibited by Ad-shERRγ **(Fig 2E)**. Consistent with the fibrinogen mRNA level, the ACEA-induced increase in the fibrinogen protein level in Huh7 cells was decreased by Ad-shERRγ **(Fig 2F)**. The fibrinogen levels in cell culture medium were increased 1.5-fold after ACEA treatment, and this effect was significantly diminished following knockdown of ERRγ by Ad-shERRγ **(Fig 2G)**. These results suggest that ERRγ acts downstream of ACEA to mediate induction of fibrinogen gene expression.

### ERRγ activates the fibrinogen gene promoter

**Fig 3:**
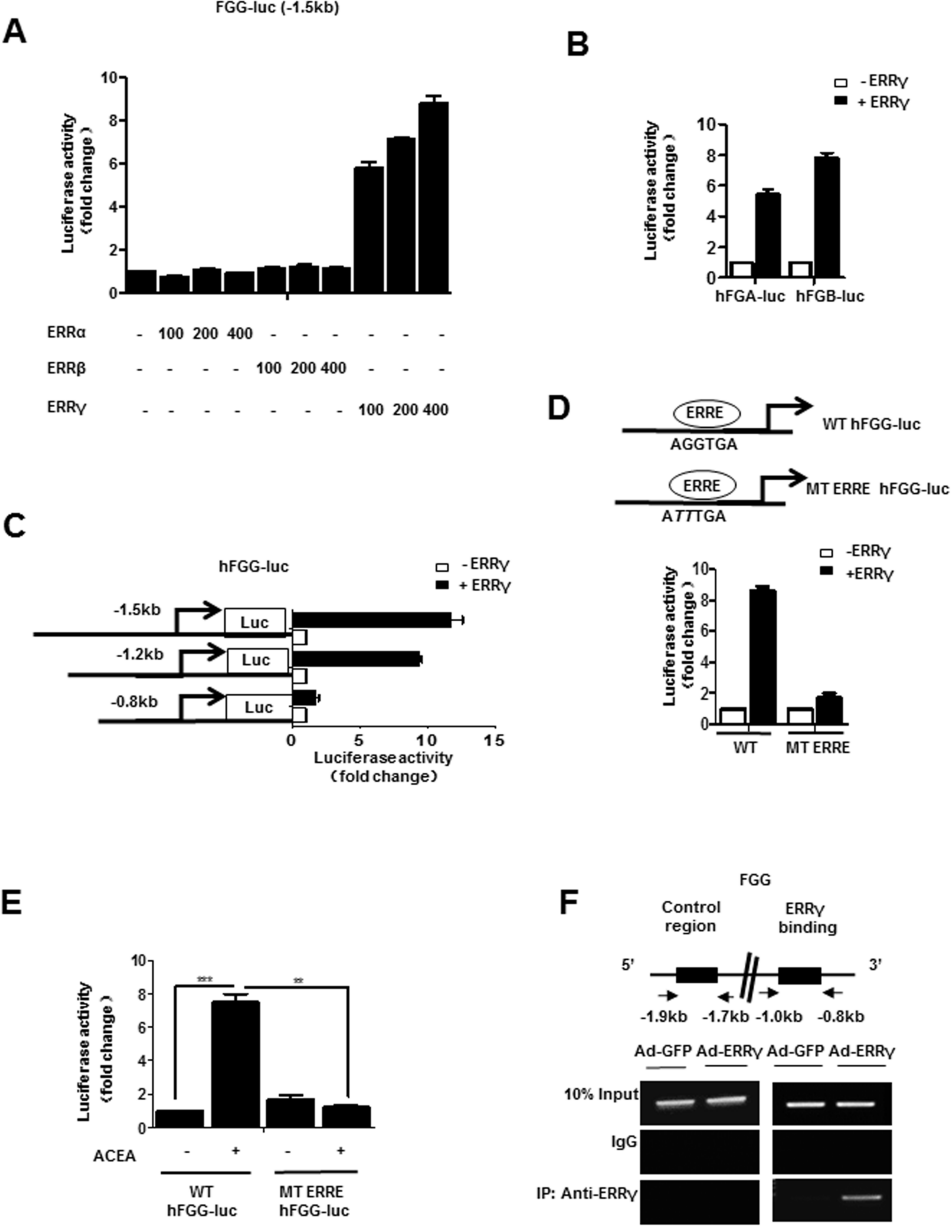
ERRγ directly regulates fibrinogen gene transcription. **(A)** ERRγ-specific induction of human FGG promoter activity. 293T cells were transfected with vectors expressing human FGG-luc and ERRα, ERRβ, and ERRγ. **(B)** ERRγ induced human *FGA* and *FGB* promoters. 293T cells were transfected with vectors expressing human FGA-luc or FGB-luc and ERRγ. **(C)** Mapping of the human *FGG* promoter. 293T cells were transfected with deletion constructs of hFGG-luc and ERRγ. **(D)** ERRE-dependent activation of the human *FGG* promoter. 293T cells were transiently transfected with pCDNA3-FLAG-ERRγ, hFGG-luc (WT), hFGG-Luc (MT ERRE). **(E)** ERRE is required for ACEA-mediated activation of the human *FGG* promoter. Huh7 cells were transfected with vectors expressing hFGG-luc (WT) or hFGG-luc (MT ERRE) and treated with ACEA (10 μM) at 36 h post transfection. Experiments in **A-E** were conducted in triplicate, and data are expressed as fold activation relative to the control. * *p*<0.05, ** *p*<0.01, *** *p*<0.001. **(F)** ChIP assay showing occupancy of the ERRE from the human *FGG* promoter by ERRγ. Huh7 cells were infected with Ad-GFP or Ad-ERRγ for 48 h. Input represents 10% of purified DNA in each sample. Cell extracts were immunoprecipitated with IgG or ERRγ antibody, and purified DNA samples were employed for PCR with primers encompassing the ERRE (-1.0 kb to -0.8 kb) and a distal site (-1.9 kb to -1.7 kb) of the *FGG* gene promoter. Error bars show SEM. The gel images were cropped with a black cropping line. Full uncropped gels are available as S3 Fig. All gels for ChIP analysis were run under the same experimental conditions.

To determine the molecular mechanism by which ERRγ regulates fibrinogen gene transcription, we transfected the luciferase reporter construct driven by the human fibrinogen promoter into 293T cells. First, we examined the effect of the ERR subfamily on the human fibrinogen promoter. ERRγ specifically increased *FGG* promoter activity in a dose-dependent manner, whereas ERRα and ERRβ had no significant effect on the *FGG* promoter **(Fig 3A)**. Further, co-transfection with ERRγ strongly induced *FGA* and *FGB* promoter activity **(Fig 3B).** In an approach using reporter assays with serial deletion constructs, we identified the DNA motif for the ERRγ effect to the region from -0.8 kb to -1.2 kb of the human *FGG* promoter, which upon deletion led to a marked decrease in ERRγ-mediated activation of the *FGG* promoter **(Fig 3C)**. Moreover, close investigations of the human *FGG* promoter revealed a putative ERRγ binding motif (AGGTGA, as indicated by ERRE). To verify ERRγ binding site in the human *FGG* promoter, we performed transfection assays using the wild type and a promoter harboring a point mutation in the putative ERRγ binding site (ERRE). Activation of the human *FGG* promoter by ERRγ was significantly abolished by the ERRE mutation **(Fig 3D)**. Human *FGG* promoter activity was increased more than 7-fold by ACEA treatment, whereas the ERRE-mutated promoter was unaffected **(Fig 3E)**. These results suggest that ERRγ directly regulates fibrinogen gene transcription through the ERRE in the human promoter. Furthermore, binding of ERRγ to the endogenous *FGG* promoter was confirmed by ChIP assays with a specific antibody against ERRγ in Huh7 **(Fig 3F)**. ERRγ was strongly recruited to the ERRE region of human *FGG* promoter, whereas no significant recruitment of ERRγ was observed in the control region. Overall, these results indicate that ERRγ directly binds and activates the fibrinogen gene promoter.

### An inverse agonist of ERRγ inhibits fibrinogen gene expression in human hepatoma cell

**Fig 4:**
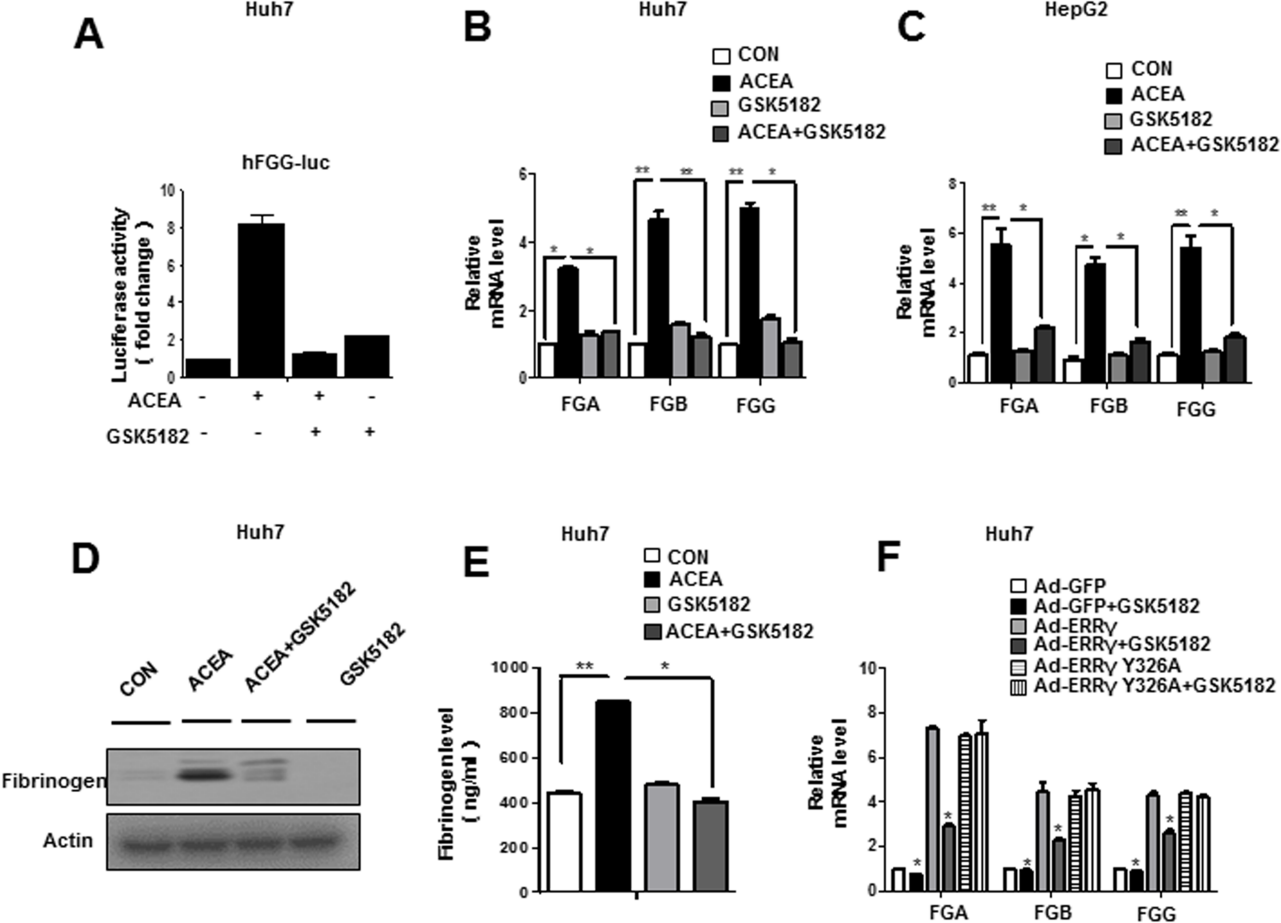
Inverse agonist of ERRγ inhibits ACEA-mediated fibrinogen gene expression in Huh7 cells. **(A)** GSK5182 decreased ACEA-mediated FGG promoter activity. Huh7 cells were transfected with vectors expression hFGG-luc, and then treated with ACEA (10 μM) and/or GSK5182 (10 μM). **(B-E)** GSK5182 inhibited ACEA-mediated fibrinogen expression and secretion in human hepatoma cell line. Huh7 and HepG2 cells were treated with ACEA (10 μM) for 12 h. The cell culture medium was replaced, and GSK5182 (10 μM) was added for the final 24 h. Total mRNA and protein were extracted for qPCR (B-C) and western blot analyses (D). Cell culture media were collected to determine fibrinogen levels in Huh7 cells (E). **(F)** GSK5182 specifically inhibits ERRγ transcriptional activity. Huh7 cells were infected with Ad-GFP, Ad-ERRγ, or Ad-ERRγ Y326A and then treated with GSK5182 (10 μM) for 24 h. Fibrinogen mRNA levels were analyzed by qPCR. * *p*<0.05, ** *p*<0.01. All data are representative of at least three independent experiments. Western blot images were cropped with a black cropping line. All gels for Western blot analysis were run under the same experimental conditions. Full uncropped blots are available as S2 Fig. Error bars show SEM.

GSK5182, an ERRγ inverse agonist, has been used to selectively inhibit transactivation byERRγ[16]. To further clarify the role of ERRγ in CB1R-mediated induction of fibrinogen gene expression, cells were transfected with the fibrinogen promoter reporter construct and treated with ACEA in the presence or absence of GSK5182. ACEA-activated human *FGG* promoter activity **(Fig 4A)** and fibrinogen gene expression **(Fig 4B and 4C)** were inhibited by GSK5182 in Huh7 and HepG2 cells. Consistent with the change in fibrinogen mRNA level, ACEA-induced fibrinogen protein levels were also significantly decreased by GSK5182 in Huh7 **(Fig 4D)**. In addition, fibrinogen levels in culture medium were dramatically increased after ACEA treatment, and this increase was significantly attenuated by GSK5182 treatment of Huh7 **(Fig 4E)**. Finally, ERRγ-mediated induction of fibrinogen mRNA was significantly decreased by GSK5182 treatment in Huh7 cells **(Fig 4F)**. Our previous results suggest that GSK5182 cannot bind to the ERRγ Y326A mutant [28]. Here, the effect of GSK5182 was abolished in the ERRγ Y326A mutant suggesting that GSK5182 specifically inhibits ERRγ transcriptional activity, eventually leading to reduced fibrinogen expression **(Fig 4F)**. Taken together, these results indicate that inactivation of ERRγ with the inverse agonist GSK5182 decreases CB1 receptor–mediated fibrinogen gene expression.

### Obese patients present with increased ERRγ and fibrinogen gene expression

**Fig 5:**
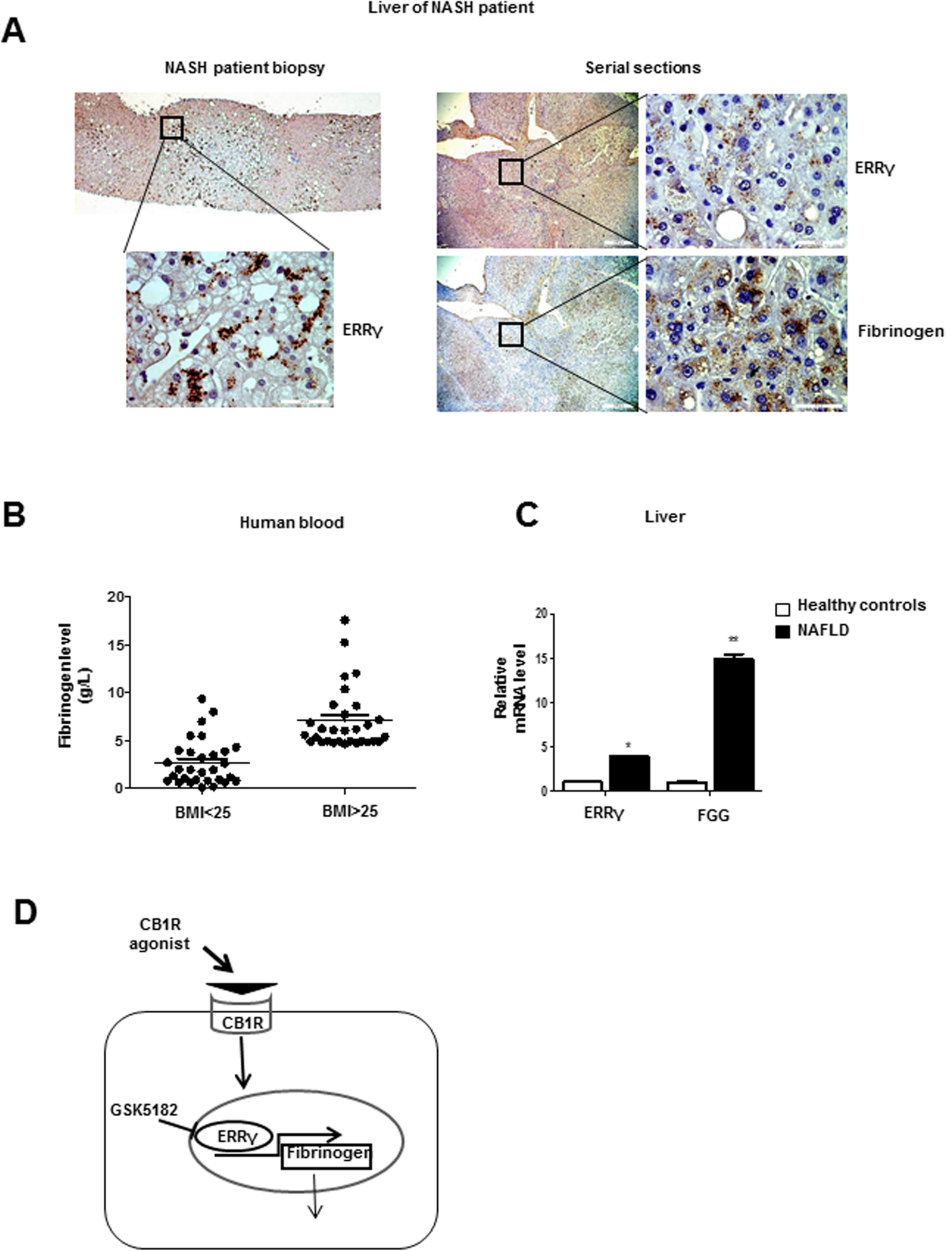
Patients with NAFLD/NASH exhibit elevated ERRγ and fibrinogen expression in the liver. **(A)** Left: representative immunohistochemistry results for ERRγ expression in liver tissue, as identically found in all three analysed patients with NASH. Right: serial sections of steatotic liver tissue showing colocalization of ERRγ and Fibrinogen staining. **(B)** Fibrinogen levels in blood of healthy controls and overweight patients. **(C)** qPCR analysis showing mRNA levels of hepatic fibrinogen gamma and ERRγ in liver tissue of healthy controls and patients with NAFLD. * *p*<0.05, ** *p*<0.01. **(D)** Proposed model for CB1 receptor–mediated induction of fibrinogen gene expression via ERRγ. Activation of hepatic CB1 receptor increases ERRγ gene expression, which in turn leads to fibrinogen expression causing hyperfibrinogenemia. GSK5182, an ERRγ inverse agonist, inhibits CB1 receptor–mediated fibrinogen gene expression.

To translate the findings in vitro to human patients with fatty liver disease, we assessed ERRγ expression by immunohistochemistry and found a significant positive staining in three out of three nonalcoholic steatohepatitis (NASH) patients, where one representative result is shown **(Fig 5A** left). Analyses of serial sections from steatotic liver areas in addition indicate coregulation of ERRγ and Fibrinogen protein expression **(Fig 5A** right). To further investigate the association between obesity and fibrinogen levels, we analyzed liver tissues and blood samples from a larger cohort of patients with fatty liver disease and controls. Blood fibrinogen concentrations were higher in overweight subjects (BMI>25) than in subjects with normal weight (BMI<25) **(Fig 5B)**. Furthermore, expression levels of ERRγ and FGG mRNAs were significantly higher in subjects with nonalcoholic fatty liver disease (NAFLD) than in those without NAFLD **(Fig 5C)**. These results suggest that increased fibrinogen levels are related to overexpression of ERRγ in obese subjects.

## Discussion

We focused our studies about regulation of fibrinogen expression on the fibrinogen γ-chain gene, because findings from most animal and cell culture studies indicate that the fibrinogen γ-chain gene is expressed at higher levels than the α- and β-chains. However, we cannot exclude the possibility that, under certain experimental conditions or in different species, regulation of fibrinogen α and β genes is equally important. For example, we observed that fibrinogen-α mRNA level was higher than the beta- and γ-chains when ERRγ was overexpressed in Huh7 cells **(Fig 1A)**. However, ACEA treatment showed the strongest induction of fibrinogen-γ mRNA level in Huh7 **(Fig 2A)**. Although Dufour *et al*. demonstrated that ERRα and ERRγ have common direct target genes, our results show that the fibrinogen promoter is activated by ERRγ, but not ERRα or ERRβ **(Fig 3A)**, highlighting the functional complexity of the transcriptional signature of the ERR subfamily. Besides, a close investigation of FGA and FGB promoters revealed potential ERRγ binding sites as found in the FGG promoter, which could be more thoroughly investigated in future research.

Recent reports indicate that hepatic CB1 receptors play a major role in the obesity-related, weight-independent component of insulin resistance [39, 43]. Further, another study found that fibrinogen level correlates with insulin [44]. These indicate that the fibrinogen level have effects on blood glucose and diabetes. In addition, a previous study found that fibrinogen levels are significantly higher in obese 6-month-old Zucker rats than in lean age-matched controls, and that treatment with the CB1R antagonist significantly reduced fibrinogen binding in old obese Zucker rats[45]. In this study, our results show for the first time that activation of hepatic CB1R can induce fibrinogen expression via ERRγ in human hepatoma cell lines, and that knockdown ERRγ by Ad-shERRγ or the inverse agonist GSK5182 can inhibit ACEA-mediated induction of fibrinogen gene expression **(Fig 4)**. These suggest that ERRγ-mediated induction of fibrinogen gene expression may be conserved in different species.

Therapeutic reduction of fibrinogen has attracted research attention since the causal link between plasma fibrinogen levels and the risk of CVD is well established, and several options for achieving this aim have been reported. Fibrinogen levels can be modulated by changes in lifestyle, of which smoking cessation is by far the most effective. Weight or stress reduction or an increase in regular physical activity may also be effective. Dietary changes appear to have a weaker effect, although regular, moderate alcohol consumption may result in a small reduction [46]. Many oral drugs decrease fibrinogen levels. Among them, the fibrates are most effective (e.g., bezafibrate reduces elevated fibrinogen by as much as 40%, and ticlopidine by about 15%) [47]. GSK5182, which is currently one of the leading ERRγ inverse agonists, shows promise for the development of drugs targeting ERRγ-related diseases. This study demonstrated that fibrinogen gene expression was reduced in a GSK5182-treated model, suggesting that this compound also provides a platform for the treatment of hyperfibrinogenemia **(Fig 4)**.

NAFLD has been traditionally regarded as the consequence of a high-fat western diet and sedentary lifestyle[48, 49]. CVD still is one major cause of mortality in NAFLD patients[48, 50, 51]. Recent research shows that of 976 inpatients and 4,742 outpatients with NAFLD, 30% had CVD or metabolic syndrome conditions and 12% presented with cirrhosis [50]. In an attempt to directly link the phenotype of NAFLD to CVD risk factors, Yeung and coworkers used metabolic nutrient overload in hepatoblastoma C3A cells and identified up-regulation of all three fibrinogen component subunits of the coagulation cascade as critical molecular link [51]. In the present study, we confirmed elevated expression of fibrinogen in liver tissue from patients with NAFLD, and that the concentration of fibrinogen correlated with BMI **(Fig 5B)**. This is consistent with the findings of the Ditschuneit group [52], who demonstrated that body weight gain increases the fibrinogen concentration and extremely overweight patients have higher fibrinogen levels, whereas weight loss is correlated with lower fibrinogen levels.

Overall, our results reveal that ERRγ plays a significant role in CB1 receptor–mediated fibrinogen gene expression. ERRγ binds to ERRE and activates human fibrinogen gene transcription. GSK5182, an ERRγ inverse agonist, resulted in a marked reduction of ERRγ-induced fibrinogen gene expression **(Fig 5D)**. The CB1R signaling pathway is activated following induction and activation of ERRγ, which plays a significant role in up-regulating fibrinogen. Therefore, targeting ERRγ represents a promising therapeutic strategy for ameliorating hyperfibrinogenemia and therewith is expected to reduce CVD risk in fatty liver patients.

## Supporting information

S1 Table Primer sequences. (PDF)

S2 Table Baseline demographic characteristics. (PDF)

S1 Fig Original (uncropped) blots of Figs 1B and 2B. (PDF)

S2 Fig Original (uncropped) blots of Figs 2F and 4D. (PDF)

S3 Fig Original (uncropped) gel of Fig 3F. (PDF)
