# Supplementary material for: Orphan nuclear receptor ERRγ is a key regulator of human fibrinogen gene expression
Source: PLoS One. 2017 Jul 27;12(7):e0182141. doi: 10.1371/journal.pone.0182141 (PMC5531639; doi:10.1371/journal.pone.0182141)
Supplement: S1 Table — (PDF) [file pone.0182141.s001.pdf]

S1 Table. Primer sequences

Name	Sequence(5'-3')
**h-FGA F**	CGTCTGCCTGGTCCTAAGTG
**h-FGA R**	TGTCTTTCCACAACCCTTGG
**h-FGB F**	CATTAGCTCCTTCCTATCTATATCCAA
**h-FGB R**	GTGAAGGGAACAGGAAATGG
**h-FGG F**	GACGCTGCTACTTTGAAGTCC
**h-FGG R**	TGGATTTGCACCGTGTCTTTG
**h-ERRγ F**	GCCCTCACTACACTGTGTGAC
**h-ERRγ R**	CCTGCTAATTTGGACTGGTCTT-3
**h-FV F**	GAGCAGGAAAGGAAGCATGT
**h-FV R**	TAGCTGTGCCGCTTCTGTC
**h-FVII F**	CTGCAGTGGGAGGACCTG
**h-FVII R**	TTGTCACCTCCCCATTTCTC
**h-FVIII F**	AGCTGGCCAGACTTCATTATTC
**h-FVIII R**	CATTGGTGCCAACAGATCC
**h-FIX F**	GATCATGGCAGAATCACCAG
**h-FIX R**	AGAATTTTGTTGGCGTTTTCA
**h-FX F**	AGATTCAAGGTGAGGGTAGGG
**h-FX R**	GACCACCTCCACCTCGTG
**h-FXI F**	ACGTCCACTGCCACTTTTTC
**h-FXI R**	GTGTCCCTGTTTGGGTGTG
**h-FXII F**	TAGGCAGCTGGACCAACG
**h-FXII R**	GGTGGAATCGAAAGTGTTGAC
**h-FXIIIB F**	TTGAAAAACCTGACTTTTATCATCA
**h-FXIIIB R**	GAAAACCACAGGGTTTCTCTTC
**Chip-FGG-CON F**	ATGGATAATGGAACTCAAGG
**Chip-FGG-CON R**	TCCGAGCCTTGTAGTGTCAG
**Chip-FGG-ERRE F**	CTCCTGCCACAGCCACAGAT
**Chip-FGG-ERRE R**	TGGAGGCTGTGTGATGCAAC
