# Supplementary material for: Orphan nuclear receptor ERRγ is a key regulator of human fibrinogen gene expression
Source: PLoS One. 2017 Jul 27;12(7):e0182141. doi: 10.1371/journal.pone.0182141 (PMC5531639; doi:10.1371/journal.pone.0182141)
Supplement: S2 Table — (PDF) [file pone.0182141.s002.pdf]

S2 Table. Baseline demographic characteristics

Parameters	Normal weight (BMI<25 kg/m²) N=30	Over weight (BMI≥25 kg/m²) N=30	*P*
**BMI (kg/m²)**	24.21±0.55	29.22±3.14	<0.001
**Age (years)**	56.67±5.55	55.47±6.73	0.454
**Gender (female, n, %)**	23(76.67)	24(80.00)	0.754
**Weight (kg)**	63.48±6.23	72.24±11.57	<0.001
**Waist circumference (cm)**	90.06±4.59	96.02±7.78	<0.001
**SBP (mmHg)**	127.22±15.81	133.00±13.50	0.133
**DBP (mmHg)**	76.06±7.67	80.63±7.99	0.027
**FPG (mmol/l)**	5.71±0.54	5.73±0.54	0.864
**TG (mmol/l)**	2.02±0.85	1.97±1.27	0.866
**TC (mmol/l)**	5.57±0.85	5.92±1.07	0.170
**LDL-c (mmol/l)**	3.68±0.78	4.02±1.06	0.159
**HDL-c (mmol/l)**	1.29±0.27	1.26±0.22	0.656
**ALT (IU/L)**	23.60±7.75	27.57±14.2	0.187
**AST (IU/L)**	23.17±4.47	25.33±5.94	0.116
**GGT (IU/L)**	30.67±17.21	40.77±25.78	0.080

Data are presented as means ± SD. *P*-value is the difference between two groups using student-*t* test or χ^2-test.

BMI: Body mass index; SBP: Systolic blood pressure; DBP: Diastolic blood pressure; FPG: Fasting plasma glucose; TG: Triglyceride; TC: Total cholesterol ; LDL-c :Low-density lipoprotein cholesterol; HDL-c: High-density lipoprotein cholesterol; ALT: Alanine transaminase; AST: Aspartate transaminase; GGT: Gamma-glutamyltransferase.
