# Supplementary figures and images for: Orphan nuclear receptor ERRγ is a key regulator of human fibrinogen gene expression
Source: PLoS One. 2017 Jul 27;12(7):e0182141. doi: 10.1371/journal.pone.0182141 (PMC5531639; doi:10.1371/journal.pone.0182141)

**S1 Fig.** Original (uncropped) blots of Figs 1B and 2B.

**1B**

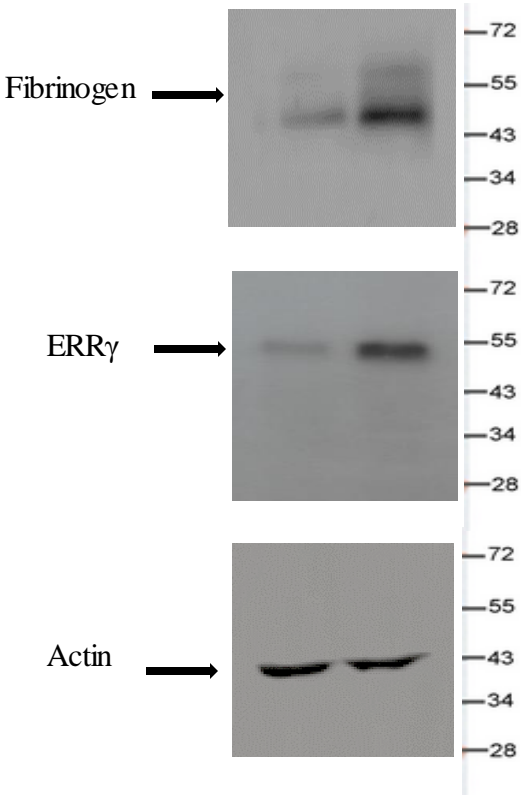

**2B**

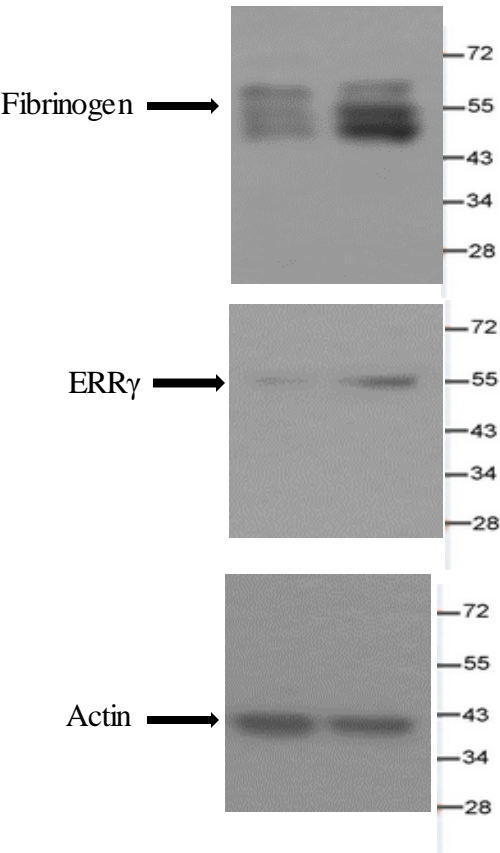

Supplement: S1 Fig — (PDF) [file pone.0182141.s003.pdf]

**S2 Fig.** Original (uncropped) blots of Figs 2F and 4D.

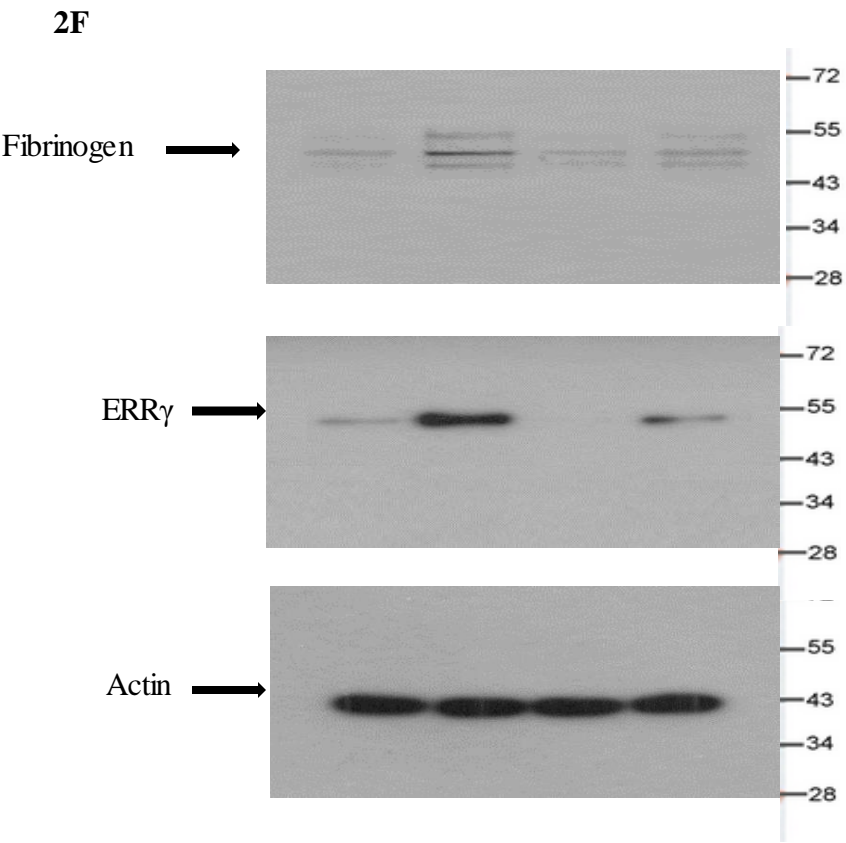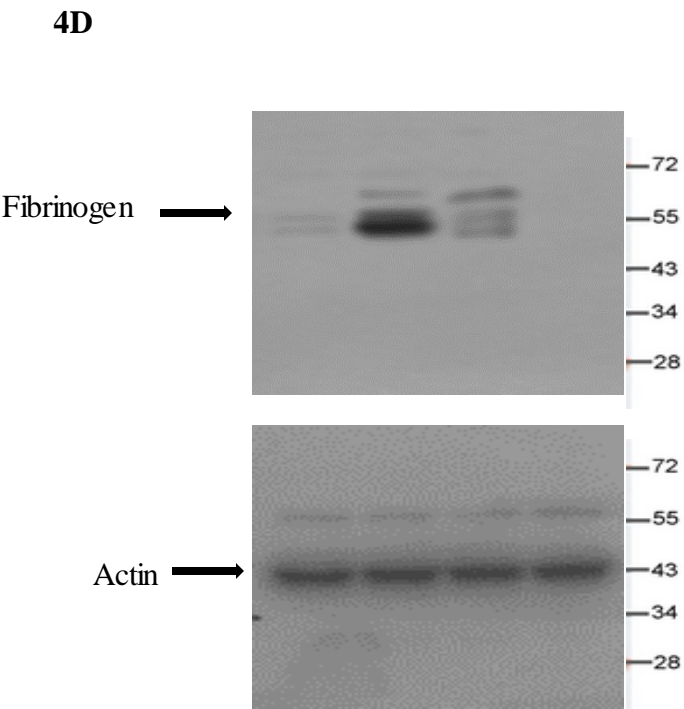

Supplement: S2 Fig — (PDF) [file pone.0182141.s004.pdf]

**S3 Fig.** Original (uncropped) gel of Fig 3F.

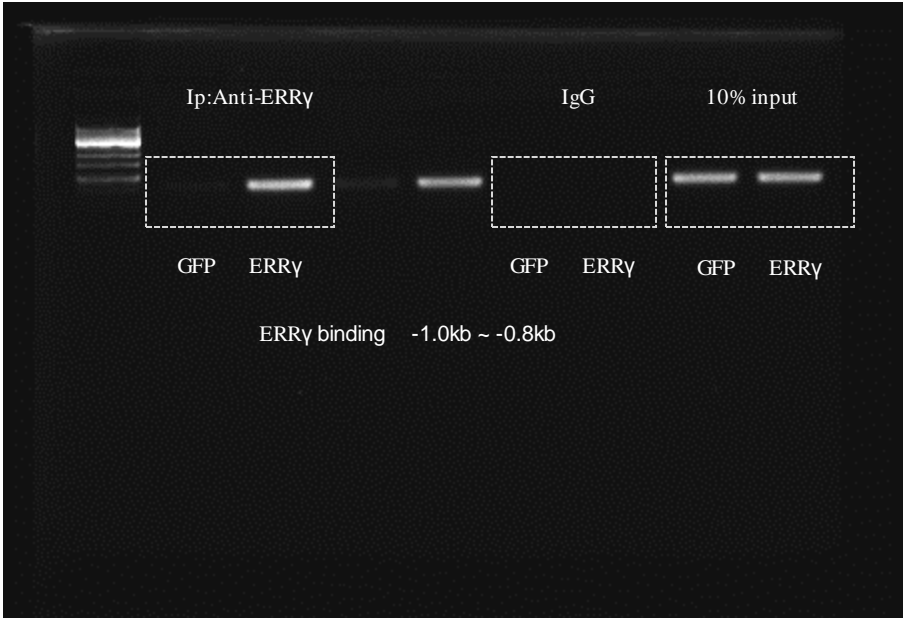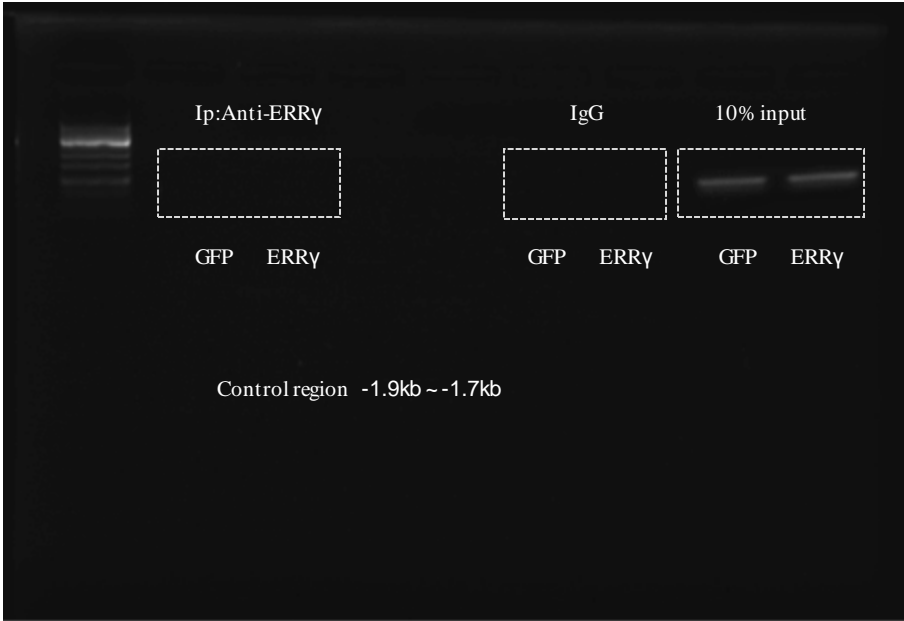

Supplement: S3 Fig — (PDF) [file pone.0182141.s005.pdf]
